# ﻿Checklist of the caddisflies (Insecta, Trichoptera) of the Upper Midwest region of the United States

**DOI:** 10.3897/zookeys.1111.72345

**Published:** 2022-07-11

**Authors:** David C. Houghton, R. Edward DeWalt, Todd Hubbard, Kurt L. Schmude, Jeffrey J. Dimick, Ralph W. Holzenthal, Roger J. Blahnik, James L. Snitgen

**Affiliations:** 1 Department of Biology, Hillsdale College, 33 East College Street, Hillsdale, MI 49242, USA; 2 Illinois Natural History Survey, 1816 South Oak Street, Champaign, IL 61820, USA; 3 State Hygienic Laboratory, University of Iowa, 2490 Crosspark Road, Coralville, IA 52241, USA; 4 Department of Natural Sciences, Lake Superior Research Institute, University of Wisconsin-Superior, 801 North 28; 5 th; 6 Street, Superior, WI 54880, USA; 7 Aquatic Biomonitoring Laboratory, College of Natural Resources, University of Wisconsin, Stevens Point, WI 54481, USA; 8 Department of Entomology, University of Minnesota, 219 Hodson Hall, 1980 Folwell Ave., Saint Paul, MN 55108, USA; 9 Environmental Health, Safety, and Land Division, Oneida Nation, P.O. Box 365, Oneida, WI 54155, USA

**Keywords:** Caddisfly, checklist, diversity, Midwest, Trichoptera, USA

## Abstract

Five hundred and fifty-two caddisfly species are reported from the Upper Midwest region of the United States, an area that includes 13 states and ~ 2 million km^2^. Of these, 62 species are reported for the first time from the state of Iowa, 25 from Wisconsin, 18 from South Dakota, 12 from Illinois, five from Indiana, four from North Dakota, four from Minnesota, and one from Nebraska. The Upper Midwest fauna contains nearly 40% of all species known from the United States and Canada, as well as 22 species endemic to the region. Overall species richness was highest in Michigan (319 species), Kentucky (296), Minnesota (292), and Wisconsin (284). Differences in state species assemblages within the region largely followed a geographic pattern, with species richness declining in the western prairie states. There are almost certainly further species remaining to be found in this large region.

## ﻿Introduction

**Figure 1.**
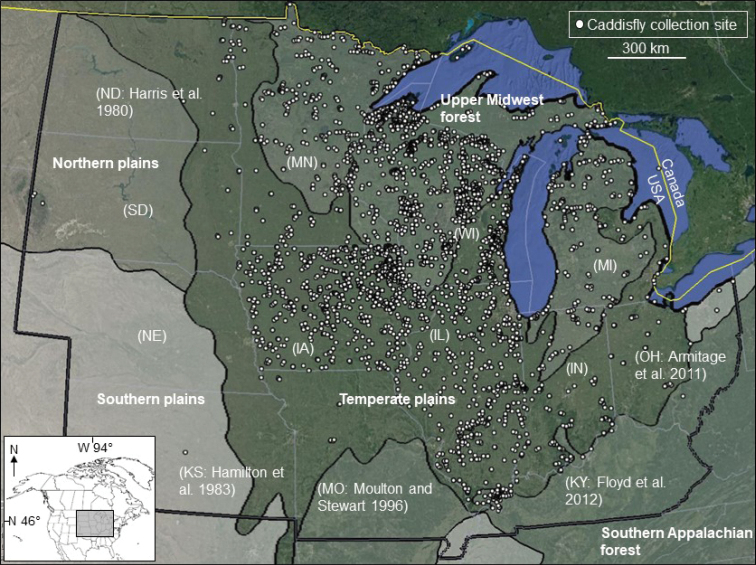
The 13 states and primary ecoregions of the Upper Midwest region, showing collecting localities within the last ~20 years by the authors or their colleagues. Citations are the most comprehensive taxonomic works for states where our collecting effort was low. State abbreviations, IA: Iowa, IL: Illinois. IN: Indiana, KS: Kansas, KY: Kentucky, MI: Michigan, MN: Minnesota, MO: Missouri, NE: Nebraska, ND: North Dakota, OH: Ohio, SD: South Dakota, WI: Wisconsin.

The Upper Midwest region of the United States (Fig. 1) encompasses 13 states and over 2 million km^2^ and is derived based on membership in the Midwest Association of Wildlife and Fisheries Agencies (MAFWA 2021). The region has a > 70-year caddisfly research history. Many of the first investigations were by Ross (1938, 1944) on the species of Illinois. Subsequent checklists on the faunas of Indiana (Waltz and McCafferty 1983), Kansas (Hamilton et al. 1983), Kentucky (Resh 1975), Michigan (Leonard and Leonard 1949), Minnesota (Etnier 1965; Houghton et al. 2001), North Dakota (Harris et al. 1980), and Wisconsin (Longridge and Hilsenhoff 1973) followed thereafter. More recently, discoveries of new records, updated checklists, and more comprehensive faunal studies have occurred in Indiana (DeWalt et al. 2016; Bolton et al. 2019), Kentucky (Floyd et al. 2012; Evans et al. 2017), Michigan (DeWalt and South 2015; Houghton 2016, 2020; Houghton et al. 2018), Minnesota (Houghton 2012), Missouri (Moulton and Stewart 1996), Ohio (Armitage et al. 2011; Bolton et al. 2019), and Wisconsin (Hilsenhoff 1995). Conversely, the caddisflies of Iowa, Nebraska, and South Dakota are known only from regional studies (Blinn et al. 2009; Zuellig et al. 2012) and piecemeal collections. Despite the extensive collecting history, new records continue to be found in the region, even in well-collected states like Michigan (Houghton 2020). The purpose of this paper was to combine historical records and our own unpublished data into a checklist of the entire Upper Midwest region, focusing on new state records and species endemic to the region.

## ﻿Materials and methods

We have been investigating the caddisflies of the Upper Midwest for ~ 20 years (Fig. 1). Collecting methods for adults have included sweep netting, malaise trapping, and ultraviolet light trapping. Most adult collecting took place during June and July, the peak emergence period of caddisflies in the region (Houghton 2018). Additional collections of adults were made during May, August, and September to obtain early and late emerging species. Larval collecting methods have included dip-netting, Hess sampling, Surber sampling, Hester-Dandy artificial substrate sampling, and hand collecting of specimens. We also accessed and confirmed specimens from the extensive Iowa (https://programs.iowadnr.gov/bionet/) and Wisconsin (https://dnr.wisconsin.gov/topic/SurfaceWater/SWIMS) Departments of Natural Resources larval macroinvertebrate databases.

Adult specimens were identified using Ross (1944), Houghton (2012), or more specific taxonomic treatments as necessary. Larvae were identified to the genus level using Morse et al. (2019 or earlier editions) and more specific species treatments as needed. Specimens collected by the authors are primarily deposited in the Hillsdale College Insect Collection, the Illinois Natural History Survey, the University of Iowa State Hygienic lab, and the University of Minnesota Insect Collection.

We also utilized the distributional checklist of Rasmussen and Morse (2020) as a starting point to investigate the presence of species that we did not personally identify. We generally accepted literature records, although we scrutinized each record for dubious assertions due to suspected misidentifications, misinterpretations of cited records, and an inability to locate the confirming specimen. Since a large portion of the Upper Midwest caddisfly checklist can already be found in Rasmussen and Morse (2020) or elsewhere, we do not recreate the entire list in this paper, but instead provide it as a supplementary data file. Nomenclature follows that of Rasmussen and Morse (2020).

Differences in caddisfly assemblages relative to geography were examined with a non-metric multidimensional scaling (NMDS) ordination using the program PC-ORD v. 7 for Windows (Peck 2016). The data matrix consisted of presence (‘1’) or absence (‘0’) values for each species for each state. All species were weighted equally. The NMDS ordination was conducted using the default program settings, 250 randomized runs, and a Jaccard distance measure. A Monte Carlo test was conducted on each determined axis to assess its difference from a random ordination structure (Dexter et al. 2018).

## ﻿Results

Based on examination of ~ 750,000 larval and adult specimens from nearly 4,000 collecting localities (Fig. 1) and a synthesis of the literature, we report 552 caddisfly species from the Upper Midwest, representing 21 families and 97 genera (Suppl. material 1). Of these, 131 species are reported for the first time from one or more states of the region (Table 1), including 62 from Iowa, 25 from Wisconsin, 18 from South Dakota, 12 from Illinois, five from Indiana, four from North Dakota, four from Minnesota, and one from Nebraska. More detailed collecting data about these species records are available in Suppl. material 2.

**Table 1.** The 131 new state species records reported herein. Species organized by family and genus. More detailed collecting data are available in Suppl. material 2.

Taxon	IA	IL	IN	MN	ND	NE	SD	WI
BRACHYCENTRIDAE								
*Brachycentrusfuliginosus* Walker, 1852	–	–	–	–	–	–	–	X
*B.lateralis* (Say, 1823)	X	–	–	–	–	–	–	–
*B.numerosus* (Say, 1823)	X	–	–	–	–	–	–	–
GLOSSOSOMATIDAE								
*Agapetustomus* Ross, 1941	–	–	–	–	–	–	–	X
*Glossosomaparvulum* Banks, 1904	–	–	–	–	–	–	X	–
*Protoptilaerotica* Ross, 1938	X	–	–	–	–	–	–	–
Helicopsychidae								
*Helicopsycheborealis* (Hagen, 1861)	X	–	–	–	–	–	–	–
Hydropsychidae								
*Cheumatopsycheaphanta* Ross, 1938	–	–	–	–	–	–	X	–
*C.campyla* Ross, 1938	–	–	–	–	–	–	X	–
*C.halima* Denning, 1948	X	–	–	–	–	–	–	–
*C.lasia* Ross, 1938	–	–	–	–	–	–	X	–
*C.minuscula* (Banks, 1907)	–	X	–	–	–	–	–	–
*C.oxa* Ross, 1938	X	–	–	–	–	–	–	–
*C.pasella* Ross, 1941	X	–	–	–	–	–	–	–
*Diplectronamodesta* Banks, 1908	X	–	–	–	–	–	–	–
*Homoplectradoringa* (Milne, 1936)	–	X	–	–	–	–	–	–
*Hydropsycheaerata* Ross, 1938	X	–	–	–	–	–	–	–
*H.alternans* (Walker, 1852)	X	–	–	–	–	–	–	–
*H.arinale* Ross, 1938	X	–	–	–	–	–	–	–
*H.betteni* Ross, 1938	–	–	–	–	–	–	X	–
*H.dicantha* Ross, 1938	X	X	–	–	–	–	–	–
*H.hageni* Banks, 1905	X	–	–	–	–	–	–	–
*H.morosa* Hagen, 1861	–	–	–	–	–	–	X	–
*H.phalerata* Hagen, 1861	–	–	–	–	X	–	–	–
*H.scalaris* Hagen, 1861	X	–	–	–	–	–	–	–
*H.slossonae* Banks, 1905	X	–	–	–	–	–	–	–
*H.sparna* Ross, 1938	X	–	–	–	–	–	–	–
*Macrostemumcarolina* (Banks, 1909)	X	–	–	–	–	–	–	–
*Parapsycheapicalis* (Banks, 1908)	X	–	–	–	–	–	–	–
Hydroptilidae								
*Agrayleamultipunctata* Curtis, 1834	X	–	–	–	–	–	–	–
*Hydroptilaajax* Ross, 1938	–	–	–	–	–	–	X	–
*H.albicornis* Hagen, 1861	X	–	–	–	–	–	–	–
*H.ampoda* Ross, 1941	–	–	–	–	–	–	–	X
*H.angusta* Ross, 1938	–	–	–	–	X	–	X	X
*H.arctia* Ross, 1938	–	–	–	–	–	–	X	–
*H.consimilis* Morton, 1905	–	–	–	–	–	–	X	–
*H.delineata* Morton, 1905	–	–	–	–	–	–	–	X
*H.grandiosa* Ross, 1938	X	–	–	–	–	–	–	–
*H.gunda* Milne, 1936	–	X	–	–	–	–	–	–
*H.metoeca* Blickle & Morse, 1954	–	–	–	–	–	–	–	X
*H.perdita* Morton, 1905	X	–	–	–	–	–	–	–
*H.quinola* Ross, 1947	–	–	–	–	–	–	–	X
*H.scolops* Ross, 1938	–	–	X	–	–	–	–	–
*H.tusculum* Ross, 1947	–	–	–	–	–	–	–	X
*H.xera* Ross, 1938	–	–	–	–	–	–	–	X
*Neotrichiaminutisimella* (Chambers, 1873)	X	–	–	–	–	–	–	–
*N.vibrans* Ross, 1938	X	–	–	–	–	–	–	–
*Ochrotrichiaalsea* Denning & Blickle, 1972	–	–	–	–	–	–	X	–
*O.arva* (Ross, 1941)	–	–	–	–	–	–	–	X
*O.riesi* Ross, 1944	–	–	–	–	–	–	–	X
*Orthotrichiacristata* Morton, 1905	X	–	–	–	–	–	–	–
*O.curta* Kingsolver & Ross, 1961	–	–	–	–	–	–	–	X
*Oxyethiraforcipata* Mosely, 1934	X	–	–	–	–	–	–	–
*O.novasota* Ross, 1944	–	X	–	–	–	–	–	–
LEPIDOSTOMATIDAE								
*Lepidostomagriseum* (Banks, 1911)	–	X	–	–	–	–	–	–
*L.liba* Ross, 1941	X	–	–	–	–	–	–	–
*L.sommermanae* Ross, 1946	–	X	–	–	–	–	–	–
*L.togatum* (Hagen, 1861)	X	–	X	–	–	–	–	–
LEPTOCERIDAE								
*Ceracleaalagma* (Ross, 1938)	X	–	–	–	–	–	–	–
*C.alces* (Ross, 1941)	X	–	–	–	–	–	–	–
*C.ancylus* (Vorhies, 1909)	X	–	–	–	–	–	X	–
*C.cancellata* (Betten, 1934)	X	–	–	–	–	–	X	–
*C.enodis* Whitlock & Morse, 1994	X	–	–	–	–	–	–	–
*C.erratica* (Milne, 1936)	–	–	–	X	–	–	–	–
*C.maculata* (Banks, 1899)	–	–	–	–	–	–	X	–
*C.neffi* (Resh, 1974)	X	–	–	–	–	–	–	–
*C.nepha* (Ross, 1944)	X	–	–	–	–	–	–	–
*C.ophioderus* (Ross, 1938)	–	–	–	–	–	–	–	X
*C.resurgens* (Walker, 1852)	X	–	–	–	–	–	–	–
*C.spongillovorax* (Resh, 1974)	X	–	–	–	–	–	–	–
*C.transversa* (Hagen, 1861)	X	–	–	–	–	–	–	–
*Leptocerusamericanus* (Banks, 1899)	–	–	–	–	–	X	X	–
*Mystacidesinterjectus* (Banks, 1914)	X	–	–	–	–	–	–	–
*Nectopsychediarina* (Ross, 1944)	X	–	–	–	–	–	–	–
*N.exquisita* (Walker, 1852)	X	–	–	–	–	–	–	–
*N.pavida* (Hagen, 1861)	X	–	–	–	–	–	–	–
*Oecetisavara* (Banks, 1905)	–	–	–	–	–	–	X	–
*O.ditissa* Ross, 1966	–	–	–	–	–	–	–	X
*O.immobilis* (Hagen, 1861)	X	–	–	–	–	–	–	–
*O.nocturna* Ross, 1966	–	–	–	–	X	–	–	X
*O.ochracea* Curtis, 1825	X	–	–	–	–	–	–	–
*Triaenodesaba* Milne, 1935	X	–	–	–	–	–	–	–
*T.baris* Ross, 1938	X	–	–	–	–	–	–	–
*T.cumberlandensis* Etnier & Way, 1973	–	X	–	–	–	–	–	–
*T.ignitus* (Walker, 1852)	X	–	–	–	–	–	–	–
*T.marginatus* Sibley, 1926	X	–	–	–	–	–	–	–
*T.melaca* Ross, 1947	X	–	–	–	–	–	–	X
LIMNEPHILIDAE								
*Asynarchusmutatus* (Hagen, 1861)	–	–	–	–	–	–	–	X
*Chilostigmodesaeroelatus* (Walker, 1852)	–	–	–	X	–	–	–	–
*Hydatophylaxargus* (Harris, 1869)	X	–	–	–	–	–	–	–
*Ironoquiapunctatissima* (Walker, 1852)	X	–	–	–	–	–	–	–
*Limnephiluscastor* Ross & Merkley, 1952	–	–	–	–	–	–	X	–
*L.femoralis* Kirby, 1837	–	–	–	–	–	–	–	X
*Platycentropusamicus* (Hagen, 1861)	X	–	–	–	–	–	–	–
*Pseudostenophylaxuniformis* (Betten, 1934)	X	–	–	–	–	–	–	–
*Psychoglyphasubborealis* (Banks, 1924)	–	–	–	X	–	–	–	–
*Pycnopsycheguttifera* (Walker, 1852)	X	–	–	–	–	–	–	–
PHILOPOTAMIDAE								
*Chimarraaterrima* Hagen, 1861	X	–	–	–	–	–	–	–
*C.obscura* (Walker, 1852)	X	–	–	–	–	–	–	–
*Dolophilodesdistincta* (Walker, 1852)	–	X	–	–	–	–	–	–
*Wormaldiamoesta* (Banks, 1914)	X	–	–	–	–	–	–	–
*W.shawnee* (Ross, 1938)	–	–	X	–	–	–	–	X
PHRYGANEIDAE								
*Agrypniastraminea* Hagen, 1873	–	–	X	–	–	–	–	–
*A.vestita* (Walker, 1852)	X	–	–	–	–	–	–	–
*Oligostomispardalis* (Walker, 1852)	–	–	–	–	–	–	–	X
*Ptilostomisangustipennis* (Hagen, 1873)	–	X	–	–	–	–	–	–
Polycentropodidae								
*Cernotinaspicata* Ross, 1938	–	–	–	X	–	–	–	X
*Holocentopusmelanae* Ross, 1938	–	–	–	–	–	–	–	X
*H.picicornis* (Stephens, 1836)	–	–	–	–	X	–	–	–
*Neureclipsispiersoni* Frazer & Harris, 1991	–	X	X	–	–	–	–	–
*Nyctiophylaxmoestus* Banks, 1911	–	–	–	–	–	–	X	–
*Plectrocnemiaalbipuncta* Banks, 1930	–	–	–	–	–	–	–	X
*P.clinei* Milne, 1936	–	–	–	–	–	–	–	X
*P.icula* (Ross, 1941)	–	–	–	–	–	–	–	X
*Polycentropuscentralis* Banks, 1914	X	–	–	–	–	–	–	–
*P.confusus* Hagen, 1861	X	–	–	–	–	–	–	–
Psychomyiidae								
*Psychomyiaflavida* Hagen, 1861	X	–	–	–	–	–	–	–
Rhyacophilidae								
*Rhyacophilavibox* Milne, 1936	X	–	–	–	–	–	–	–
Thremmatidae								
*Neophylaxayanus* Ross, 1938	–	X	–	–	–	–	–	–
**Total**	62	12	5	4	4	1	18	25

**Figure 2.**
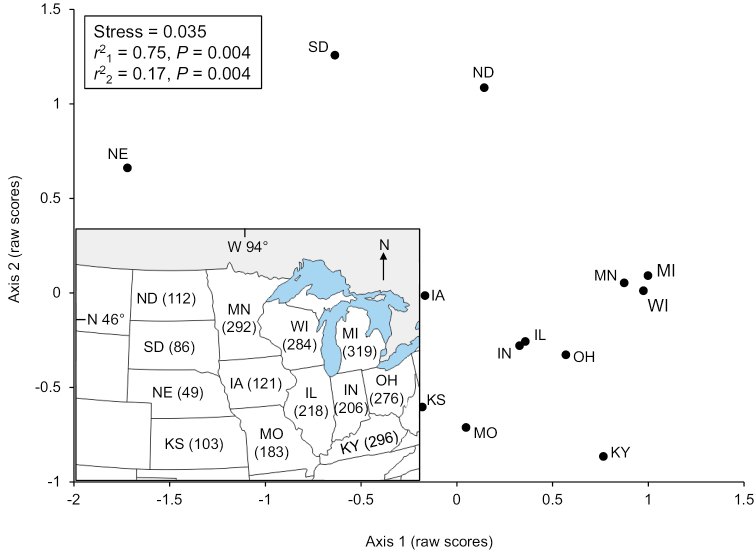
The 13 states of the Upper Midwest region delineated by location and by the results of an NMDS ordination of caddisfly species presence or absence per state. Total number of species for each state in parentheses. State abbreviations in Fig. 1.

Michigan (319) had the greatest species richness, followed by Kentucky (296), Minnesota (292), and Wisconsin (284) (Fig. 2). Only 13 species (2%) were found in all Upper Midwest states, whereas 144 species (26%) were found in a single state (Suppl. material 1). Of these single-state species, 53 (37%) were found exclusively in Kentucky and 21 (15%) in Missouri. A total of 22 species are reported as regional endemics (Table 2).

**Table 2.** The 22 species that are global endemics to the Upper Midwestern region, organized by family and genus, and with known number of collection localities and recent collection year. Superscript references are after the table.

Taxon	IL	KY	MI	MN	MO	ND	NE	OH	No. localities	Collected
GLOSSOSOMATIDAE										
*Agapetusartesus* Ross, 1938	–	–	–	–	X	–	–	–	3	2017^a^
*Protoptilatalola* Denning, 1948	–	–	–	X	–	–	–	–	1	1941^b^
Hydroptilidae										
*Hydroptiladanieli* Harris & Armitage, 2011	–	–	–	–	–	–	–	X	6	1998^c^
*H.howelli* Houp, Houp & Harris, 1998	–	X	–	–	–	–	–	–	3	1998^d^
*H.kuehnei* Houp, Houp, & Harris, 1998	–	X	–	–	–	–	–	–	5	1998^d^
*H.paraxella* Harris & Armitage, 2011	–	X	–	–	–	–	–	X	3	2008^c^
*Neotrichiaparaokopa* Keth, 2015	–	–	–	–	X	–	–	–	1	2013^d^
*N.staufferi* Keth, 2015	X	–	–	–	–	–	–	–	1	2013^d^
*Oxyethiraitascae* Monson & Holzenthal, 1993	–	–	X	X	–	–	–	–	~20	2014^e^
LEPTOCERIDAE										
*Ceracleabrevis* (Etnier, 1968)	–	–	–	X	–	–	–	–	1	1965^b^
*C.erulla* (Ross, 1938)	–	–	–	–	–	–	–	X	1	1930s^b^
*C.maccalmonti* Moulton & Stewart, 1992	–	–	–	–	X	–	–	–	2	2002^f^
*Setodestruncatus* Houghton 2021	–	–	X	–	–	–	–	–	2	2019^e^
*Triaenodesphalacris* Ross, 1938	–	–	–	–	–	–	–	X	1	1930s^b^
LIMNEPHILIDAE										
*Chilostigmaitascae* Wiggins 1975	–	–	–	X	–	–	–	–	4	2020^e^
*Glyphopsychemissouri* Ross, 1944	–	–	–	–	X	–	–	–	2	2017^a^
*Ironoquiaplattensis* Alexander & Whiles, 2000	–	–	–	–	–	–	X	–	~25	2013^g^
Polycentropodidae										
*Cernotinaohio* Ross, 1939	–	–	–	–	–	–	–	X	1	1930s^b^
*Holocentropuschellus* (Denning, 1964)	–	–	–	–	–	X	–	–	1	1960s^b^
*H.milaca* (Etnier, 1968)	–	–	X	X	–	–	–	–	6	2021^e^
*Plectrocnemiasabulosa* (Leonard & Leonard, 1949)	–	–	X	–	–	–	–	–	5	2019^e^
*Polycentropusneiswanderi* Ross, 1947	X	X	–	–	–	–	–	X	4	1990s^c,d^

^a^Mabee et al. (2019), ^b^known only from holotype, ^c^Armitage et al. (2011), ^d^Floyd et al. (2012), ^d^Armitage et al. (2015), ^e^collected by the authors, ^f^Ferro and Sites 2007, ^g^Vivian et al. 2013

The NMDS ordination of species presence or absence per state produced a two-dimensional solution (Fig. 2). The two axes reflected > 90% of variation within the dataset. Distribution of the 13 states in ordination space had a high congruence with states in geographic space.

**Figure 3.**
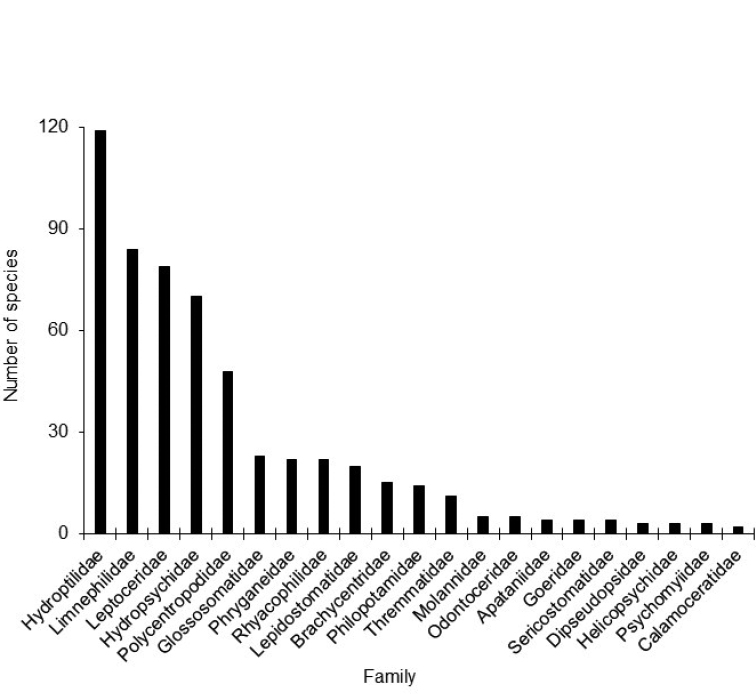
The total number of caddisfly species within each family known from the Upper Midwest region. *N* = 552 total species.

Hydroptilidae (117 species) was the most species rich family, followed by Limnephilidae (82), and Leptoceridae (76) (Fig. 3). Those families, plus the Hydropsychidae and the Polycentropodidae collectively represented nearly 75% of all species richness. The most species rich genera were *Hydroptila* (56 species), *Hydropsyche* (35), and *Limnephilus* (31) (Suppl. material 1).

## ﻿Discussion

The majority of our reported new state records are species found in at least one other Upper Midwest state. Many of these species, such as *Ceracleamaculata* (Banks) (Leptoceridae) in South Dakota or *Psychomyiaflavida* Hagen (Psychomyiidae) in Iowa, are common and widespread throughout the region. Thus, their recent discovery almost certainly reflects a lack of collecting in particular states.

Conversely, a few of our reported species represent some interesting range extensions. *Chilostigmodesaeroelatus* (Walker) (Limnephilidae) is known throughout Alaska and Canada (Rasmussen and Morse 2020), and our Minnesota collection represents the first record of the genus and species within the lower 48 states. *Limnephilusfemoralis* Kirby (Limnephilidae) is a northern Holarctic species which has recently been collected in Michigan (Houghton 2020) and Wisconsin, in addition to the states of Maine and Washington (Rasmussen and Morse 2020). *Triaenodescumberlandensis* Etnier and Way (Leptoceridae) was known only from the southeastern USA prior to our collection in Illinois. *Glossosomaparvulum* Banks (Glossosomatidae), *Ochrotrichiaalsea* Denning & Blickle (Hydroptilidae), and *Limnephiluscastor* Ross & Merkley (Limnephilidae) are all western species (Rasmussen and Morse 2020), and our records of them in western South Dakota probably represent the eastern edge of their range. *Cernotinaspicata* Ross (Polycentropodidae) was collected from both Wisconsin and Minnesota, thereby extending the known range of the species and the genus westward by nearly 800 km.

The 22 documented endemic species represent 4% of the total caddisfly fauna of the Upper Midwest. Not surprisingly, most of these species are rare and have been found at < 10 total localities throughout their ranges (Table 2). Most of the species have been collected within the last 10­–20 years. The exceptions include *Ceracleabrevis* (Etnier), *C.erulla* (Ross), *Triaenodesphalacris* Ross (Leptoceridae), *Cernotinaohio* Ross, *Holocentropuschellus* (Denning) (Polycentropodidae), and *Protoptilatalola* Denning (Glossosomatidae), all of which are known only from their respective holotypes and have not been collected in > 50 years. *Ceracleabrevis* and *P.talola* are the subjects of taxonomic uncertainty due to the similarities of their holotypes to *C.tarsipunctata* (Vorhies) and *P.tenebrosa* (Walker), respectively (Houghton 2012). The uncertainty is compounded by the poor state of the holotype specimens. The holotype for *H.chellus* is in a similarly poor state (Nimmo 1986). *Ironoquiaplattensis* Alexander & Whiles (Limnephilidae) is almost certainly the best studied of all Upper Midwest endemics. It is known from a series of locations within the Platte River drainage in Nebraska, where it appears to be decreasing in both prevalence and abundance due to drought, habitat loss, and cattle grazing (Harner and Geluso 2012; Vivian et al. 2013).

The congruence of state species assemblages with geographic location was noteworthy and probably due to a combination of factors. Both latitude and longitude have been previously shown to affect caddisfly assemblages (Moulton and Stewart 1996; Houghton 2004; Blinn and Ruiter 2013; Shah et al. 2014). While some assemblage differences in our study certainly reflect species replacement over geographic distance, a large portion of the eastern-to-western gradient was probably also due to low species richness in the western prairie states of the region, namely Kansas, Nebraska, North Dakota, and South Dakota (Fig. 2). Indeed, Nebraska has fewer known total caddisfly species (49) than what was frequently collected from a single blacklight trap in northern Minnesota, Michigan, or Wisconsin. This lower richness is probably due to a combination of the naturally arid environment of the western states (McNeely 2003), a high level of habitat degradation due primarily to agriculture (Houghton 2021), and a lack of sampling effort. Even basic species checklists have yet to be compiled for Nebraska and South Dakota. Iowa, similarly, had limited sampling effort prior to this study, and the known species richness of the state more than doubled based on the new records reported herein. Further sampling effort in the western portion of the Upper Midwest region will be needed to clarify the actual caddisfly assemblages and their correspondence with geographic location.

The total determined caddisfly species richness of the Upper Midwest region currently represents 37% of all described species from the United States and Canada, as well as 63% of genera and 81% of families (Rasmussen and Morse 2020). It is likely that many new caddisfly species remain to be discovered in the region. For example, Illinois is one of the best-collected states in both the Upper Midwest region and in the entire USA (Ross 1938; Ross 1944), and yet we found 12 new species records from the state. Future research should focus on states with minimal collecting effort, such as Nebraska and South Dakota, since these states undoubtedly still contain undiscovered caddisfly records.

## Supplementary materials

Supplementary material 1 Current checklist of 552 caddisfly species known from the Upper Midwest region Data type species data https://binary.pensoft.net/file/712624This dataset is made available under the Open Database License (http://opendatacommons.org/licenses/odbl/1.0/). The Open Database License (ODbL) is a license agreement intended to allow users to freely share, modify, and use this Dataset while maintaining this same freedom for others, provided that the original source and author(s) are credited.David C. Houghton, R. Edward DeWalt, Todd Hubbard, Kurt L. Schmude, Jeffrey J. Dimick, Ralph W. Holzenthal, Roger J. Blahnik, James L. Snitgen

Supplementary material 2 Collection data for the 131 new state species records Data type species data https://binary.pensoft.net/file/712625Explanation note: Copyright notice: This dataset is made available under the Open Database License (http://opendatacommons.org/licenses/odbl/1.0/). The Open Database License (ODbL) is a license agreement intended to allow users to freely share, modify, and use this Dataset while maintaining this same freedom for others, provided that the original source and author(s) are credited.David C. Houghton, R. Edward DeWalt, Todd Hubbard, Kurt L. Schmude, Jeffrey J. Dimick, Ralph W. Holzenthal, Roger J. Blahnik, James L. Snitgen
